# A neuromarker of individual general fluid intelligence from the white-matter functional connectome

**DOI:** 10.1038/s41398-020-0829-3

**Published:** 2020-05-13

**Authors:** Jiao Li, Bharat B. Biswal, Yao Meng, Siqi Yang, Xujun Duan, Qian Cui, Huafu Chen, Wei Liao

**Affiliations:** 1 The Clinical Hospital of Chengdu Brain Science Institute, School of Life Science and Technology, University of Electronic Science and Technology of China, Chengdu, 610054 PR China; 2 MOE Key Lab for Neuroinformation, High-Field Magnetic Resonance Brain Imaging Key Laboratory of Sichuan Province, University of Electronic Science and Technology of China, Chengdu, 610054 PR China; 3 Department of Biomedical Engineering, New Jersey Institute of Technology, Newark, NJ 07102 USA; 4 School of Public Administration, University of Electronic Science and Technology of China, Chengdu, 610054 PR China

**Keywords:** Predictive markers, Human behaviour

## Abstract

Neuroimaging studies have uncovered the neural roots of individual differences in human general fluid intelligence (*Gf*). *Gf* is characterized by the function of specific neural circuits in brain gray-matter; however, the association between *Gf* and neural function in brain white-matter (WM) remains unclear. Given reliable detection of blood-oxygen-level-dependent functional magnetic resonance imaging (BOLD-fMRI) signals in WM, we used a functional, rather than an anatomical, neuromarker in WM to identify individual *Gf*. We collected longitudinal BOLD-fMRI data (in total three times, ~11 months between time 1 and time 2, and ~29 months between time 1 and time 3) in normal volunteers at rest, and identified WM functional connectomes that predicted the individual *Gf* at time 1 (*n* = 326). From internal validation analyses, we demonstrated that the constructed predictive model at time 1 predicted an individual’s *Gf* from WM functional connectomes at time 2 (time 1 ∩ time 2: *n* = 105) and further at time 3 (time 1 ∩ time 3: *n* = 83). From external validation analyses, we demonstrated that the predictive model from time 1 was generalized to unseen individuals from another center (*n* = 53). From anatomical aspects, WM functional connectivity showing high predictive power predominantly included the superior longitudinal fasciculus system, deep frontal WM, and ventral frontoparietal tracts. These results thus demonstrated that WM functional connectomes offer a novel applicable neuromarker of G*f* and supplement the gray-matter connectomes to explore brain–behavior relationships.

## Introduction

Neuroimaging and psychological studies have investigated the neural basis of the cognitive processes that motivate novel insights about brain–behavior relationships^1^. An enduring aim of brain and cognitive sciences is to understand individual differences in human intelligence^2^. Human general fluid intelligence (*Gf*) refers to an ability to think logically and to solve novel problems that do not rely on previously acquired knowledge^3,4^. *Gf* has been broadly quantified using a series psychometric test, which further provides a foundation to process brain–behavior associations^5,6^. Given that individual differences are inherent to *Gf*, it is crucial to identify neural correlates of *Gf* and corresponding variations in brain structure and function.

The neural correlates of individual differences in *Gf* may be associated with variations in brain size and connections^2^. A larger brain size (volume) consistently indicates higher intelligence^7^; this concept of “the bigger brain, the better intelligence” may result from the efficiency of information flow among neurons^5,8^. Recently, the information flow among certain areas associated with *Gf* have been quantified by functional connectivity studies. These results showed that the variational relationships of regions engaging in common or related performance (even at rest) may be the basis of individual differences in *Gf*^9–11^, and measurements of the activity of the resting-state human brain might carry information about intelligence^12^. Furthermore, the rate of information flow among the distributed parietal and frontal areas composing the parieto-frontal integration theory (P-FIT) network are likely to play key roles in intelligence^13^. It is not surprising that a large number of these brain regions and brain functional networks are related to individual *Gf*^12,14,15^, due to the diverse abilities associated with *Gf*, including understanding of daily tasks and problem solving. Accordingly, whole-brain functional connectivity measures may provide more holistic insight to determine an individual’s *Gf*, rather than global brain size.

*Gf* is mainly rooted in the functional connectivity of specific neural circuits within gray-matter (GM); however, little is known regarding the neurofunctional substrates of individual differences in *Gf* in white-matter (WM)^16–18^. Recently, a small but increasing number of investigations have demonstrated a reliable detection of blood-oxygen-level-dependent functional magnetic resonance imaging (BOLD-fMRI) signals in WM. These studies indicate that neural activation elicits temporal and spectral profiles of hemodynamic responses in WM that are similar to those measured in GM during different functional tasks^19–24^. In parallel with the detection of task-related activations, BOLD-fMRI can also reflect the neural activity in WM at rest (i.e., absence of task requirement)^25,26^. Specifically, we found that, during the resting state, the power of low-frequency BOLD-fMRI fluctuations in WM exhibited a specific rather than a random distribution of noise^26^, and the WM functional connectome exhibited reliable and stable small-worldness and nonrandom modularity^27^. Abnormal small-worldness in the WM functional connectome was reported in patients with Parkinson’s disease^28^. Furthermore, the specific functional connectivity organization of the anatomical bundles was able to be identified by resting-state fMRI^20,21,25,29^. These investigations not only provided evidence of neural activity and connectivity, using fMRI, but also established cognitive biomarkers (i.e., for memory function) in WM^30^.

In the current study, using a cross-validation, data-driven analysis, we present novel findings that the whole-brain WM functional network predicted individual *Gf* and shed light on the possible neurofunctional correlates of *Gf* in WM. We first built a network-predicted model between connectivity strength and *Gf* scores of normal individuals following an initial examination (time 1, *n* = 326 participants). We demonstrated that the network-predicted model derived from these data could predict an individual’s *Gf* from his/her WM functional connectivity. This predictive model constructed at time 1 can be generalized to both the second (time 2, *n* = 105 participants) and the third examinations (time 3, *n* = 83 participants) using the overlapped individuals, thus, accurately predicting an individual’s *Gf* score from WM functional connectivity during the time 2 and time 3 scans for internal validation. Finally, to further test the generalizability of the predicted model, we showed that this model could also predict novel independent performance *Gf* (*n* = 53 participants) for external validation. These results suggested that the whole-brain functional connectivity of WM was a neuromarker of individual differences in *Gf* and would generalize to independent data to predict individual *Gf*.

## Materials and methods

### Participants

An overview of this study is shown in Fig. S1. Two independent cohorts were enrolled, namely, the internal and external validation groups. The interval validation group was further divided into the three subgroups. Internal validation I (time 1) was trained on time 1 data and also tested on time 1 data. Internal validation II (time 1 ∩ time 2) was trained on time 1 data (*n* − 1 participants) and tested on both time 1 and time 2 data (~11 months after time 1). Internal validation III (time 1 ∩ time 3) was trained on time 1 data (*n* − 1 participants) and tested on both time 1 and time 3 data (~29 months after time 1). All participants were normal college students from Southwest University of China, Chongqing, China. This study was approved by the Institutional Human Participants Review Board of the Southwest University Imaging Center. Written informed consent was obtained from all subjects. The data are available for research purposes through the International Data-sharing Initiative (http://fcon.1000.projects.nitrc.org/indi/retro/southwestuni/qiu/index.html). For detailed description about the participant information and data acquisition parameters, please see Liu et al.^31^.

For the external validation group, completely independent normal controls were recruited (see SI Materials and Methods for detailed information).

### Assessment of fluid intelligence

For the internal validation group, the participants’ intellectual ability was assessed by the Combined Raven’s Test (CRT) (Chinese revised version) at time 1^32^, which demonstrates a high degree of reliability and validity for intelligence testing^32,33^. This test gives an indication of the level of analogical thinking and abstract thought that a person has achieved; therefore, this test is known to be a good indicator of *Gf*^5,11,34^. The CRT scores (the number of correct answers given in 40 min) were used as a psychometric index of individual intelligence. In line with standard practice, the current study focused on the total score of the test^4,34,35^.

For the external validation group, the participants’ *Gf* abilities were assessed with performance general intelligence using the Chinese version of the Wechsler Adult Intelligence Scale (WAIS-RC). The Raven’s Test and WAIS-RC are clearly dominant in terms of interest, and are highly correlated between them in college students^36^.

### Neuroimaging data acquisition

For the internal validation group, structural and functional MRI images were collected using a Siemens Trio 3.0T scanner (Siemens Medical, Erlangen, Germany) at the Southwest University China Center for Brain imaging^31^. The T1-weighted structural images (repetition time = 1900 ms, echo time = 2.52 ms, flip angle = 9°, field of view = 256 × 256 mm^2^, matrix size = 256 × 256, voxel size = 1 × 1 × 1 mm^3^, and slices = 176) were acquired. Subsequently, resting-state fMRI were acquired using a single-shot, gradient-recalled echo planar imaging sequence (repetition time = 2000 ms, echo time = 30 ms, flip angle = 90°, field of view = 220 × 220 mm^2^, matrix size = 64 × 64, voxel size = 3.4 × 3.4 × 3 mm^3^, and slices = 32). For each participant, a total of 242 functional volumes (484 s) were acquired. All participants were instructed to simply rest with their eyes closed, and not to think of anything in particular. For the external validation group, the parameters of data acquisition are list in “Materials and methods.”

### Neuroimaging data preprocessing

Neuroimaging data were analyzed using the DPARSF (v4.3, www.restfmri.net) and SPM12 toolkits (www.fil.ion.ucl.ac.uk/spm/software/spm12). Slice-timing correction and realignment were applied to the remaining 235 functional images after excluding the first seven images. Structural images were then co-registered to the preprocessed functional images, and then segmented into GM, WM, and cerebrospinal fluid (CSF) by using DARTEL^37^. The mean signals from CSF (95% thresholded), 24 head motion parameters (six motion parameters, six temporal derivatives, and their respective squares) were regressed out from the data. To avoid elimination of important neural signals, we did not remove WM and brain global signals, as previous studies have suggested^19,26,29^.

To minimize mixing GM signals with WM signals, subsequent preprocessing for functional images was performed for WM alone. First, individual masks were obtained using a rigorous 90% threshold on the probability map of WM, which was produced by structural segmentation. Second, functional images were then spatially restricted into WM images using dot products between functional images and individual masks. Third, the WM functional images were then spatially normalized into the Montreal Neurologic Institute space by structural segmentation and were resampled into 3 × 3 × 3 mm^3^. Then, only voxels identified as WM across 80% of the participants were included into the group-level WM mask production. To exclude the impact of deep brain structures, the probability (25% threshold) Harvard-Oxford Atlas was used to remove subcortical nuclei (i.e., the bilateral thalamus, putamen, caudate, pallidum, and accumbens) from the group-level WM^28,29,38^. To minimize spurious local spatial correlations between voxels, spatial smoothing was not applied^39,40^. Subsequently, a band-pass filtering (0.01–0.10 Hz) was performed to minimize high-frequency physiological noise sources including the respiration rate. Finally, as functional connectivity is sensitive to the confounding factor of head motion, scrubbing was performed to reduce the negative influence^41^. If the framewise displacement (FD) exceeded 0.5 mm, the value of the signal at the point, as well as one forward point and two points previous to the signal point, were removed. Participants with 80% of their volumes remaining were included in further analyses.

### Quality control

Exclusion criteria in selecting research participants are shown in Fig. S2. As head motion deteriorates the quality of fMRI data, we defined a set of standards to control for head motion. Specifically, if there was translational or rotational head movement > 2 mm or 2°, respectively, or head micromovements (mean FD) were >0.15 mm during resting-state fMRI scanning, these data were excluded. In addition, following scrubbing analyses, if a participant’s points on an image were <80%, their data were excluded. The quality of functional images (e.g., whether most of the temporal lobe was not visible) were then checked from the remaining participants. Quality control was applied to participants at time 1, time 2, and time 3. Finally, for each validation group, a prior outlier was defined as greater than mean + 2 standard deviation (SD) or less than mean − 2 SD for *Gf* scores, and were excluded. Finally, 326 participants (142 females, mean age for males and females = 20.03 years, SD = 0.072 years) were included in the internal validation I group (time 1); 105 participants were included in the internal validation II group (time 1 ∩ time 2); and 83 participants were included in the internal validation III group (time 1 ∩ time 3). The demographics for each time point/sample are shown in Table S1.

### Construction of WM functional connectome

**Fig. 1:**
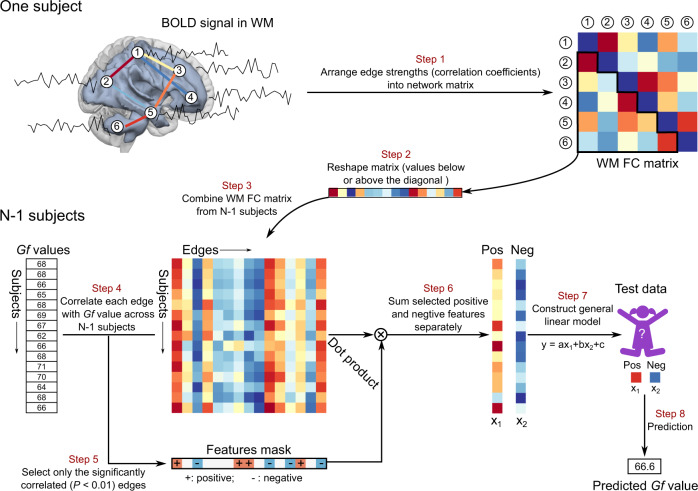
Schematic diagram of the WM functional connectivity-based predictive model. Step 1: We first obtained BOLD-fMRI signals in WM and then computed functional connectivity matrixes between each pair of nodes using Pearson’s correlation. Step 2: Each matrix values below or above the diagonal was reorganized to a row. Step 3: In each iteration, all rows were combined into a new matrix (*m* × *n*, where *m* represents the number of participants, and *n* represents the number of edges) across *n* − 1 participants (*n* is the number of participants). Step 4: Obtaining the correlation coefficients between each edge and an individual’s Gf abilities across *n* − 1 participants. Step 5: Significantly correlated edges (*P* < 0.01) with Gf was selected to construct features mask. These correlated edges were separated into a positive network (correlation coefficients were positive) or a negative network (correlation coefficients were negative). The dot product method was then applied between *m* × *n* matrix and feature masks. Step 6: Selected positive features and negative features were summed separately as two features into the predictive model (general linear model). Step 7: A general linear model was employed to construct the predictive model. Step 8: Test data matrixes were also performed using the dot product method with feature masks to obtain two features, which were added to the constructed predictive model to predict the Gf score. These steps were repeated *N* times, and Gf scores were obtained in all participants. Finally, correlation was calculated between observed and predicted Gf scores. BOLD-fMRI blood-oxygen-level-dependent functional magnetic resonance imaging, Gf general fluid intelligence.

Nodes in WM functional networks were defined using group-wise voxel-based parcellation algorithms that produce roughly equal sizes within each node^42^. To obtain the 128 nodes used in this study, the parcellation algorithm was applied to the group-level WM mask from the remaining 326 participants at time 1. This parcellation scheme, which does not rely on a prior anatomical structure, may be better than the anatomical-based parcellation scheme, because of the uncertain match qualities between anatomical and functional images^27^, and is frequently used in GM mask parcellation^40,43^. In addition, the parcellation in the WM functional mask has been reported to have stable and reliable small-worldness^27^. For each participant, the WM functional connectivity matrix (128 × 128) was calculated by Pearson’s correlation between averaged BOLD signals of paired nodes. For the connectivity matrix, each cell represented the connection (i.e., edge) strength (Fisher’s *r*-to-*Z* normalized value) of each pair of nodes (step 1 in Fig. 1)^44,45^. Connectivity matrices were not thresholded or binarized^46^.

### Predictive model for internal validation

To investigate whether the strength of WM functional connectivity predicted individual *Gf*, we employed a leave-one-out cross-validation (LOOCV) method, which avoided overfitting by testing the strength of the relationship in an unseen participant^1^. Importantly, because head micromovements may cause spurious connectivity patterns, we first identified and excluded edges sensitive to head motion^47^. In each LOOCV, we randomly selected one participant as a testing set, and the remaining *n* − 1 participants were used as a training set (step 3 in Fig. 1)^48^. The predictive features were defined as the relevant edges to *Gf* scores at a significant threshold of *P* < 0.01 in the training set (*n* − 1 participants) (steps 4 and 5 in Fig. 1). The *r* values were then separated into a positive network (where *r* values were positive) and a negative network (where *r* values were negative)^1,47,49^. For each participant in the LOOCV, we summed the strength of edges from the WM functional connectivity matrix in the positive and negative sets, separately (step 6 in Fig. 1). Positive and negative sets have been reported to have different functional roles and might separately contribute to predictive power^1,11,46^. Hence, a simplified general linear model (GLM) protocol, which combined the positive and negative networks (step 7 in Fig. 1), was used to construct the training model in each LOOCV (the positive and negative models were also constructed for exploration). To predict the left-out participant’s (defined as testing data) *Gf* score, the training model was applied to the testing data, i.e., the strength of functional positive and negative networks (step 8 in Fig. 1). The flowchart of the predictive model is shown in Fig. 1. After repeating the abovementioned procedure n times, each participant’s *Gf* score was obtained. Finally, we computed the correlation between observed and predicted *Gf* scores using Pearson’s correlation to evaluate the power of the predictive model. *K*-fold cross-validation^50^ was also used to test the relationship between observed and predicted *Gf* scores. Participants were separated into 20-fold in line with previous work^51^, and ~16 participants were included in each fold. Similar to the LOOCV procedure, onefold was left out as a testing data, and the remaining 19-fold were used as training data to construct the predictive model in each cross-validation. After repeating this procedure 20 times, we obtained *Gf* scores. To minimize the random error on folds’ partition, we repeated the above predictive procedure 50 times^52^.

### Permutation test

We first used parametric statistical analysis to obtain *P* values in the LOOCV procedure. However, the number of degrees of freedom are overestimated when LOOCV is performed within a single data set^1,46^. A permutation test is a commonly used statistical tool offering a simple way to compute the sampling distribution for any test statistic under the null hypothesis. We kept our WM functional connectivity matrices unchanged and randomly shuffled observed *Gf* scores 5000 times. The prediction procedure was then performed on each shuffled data. A distribution of the test statistic was obtained. The *P*_permutation_ value was calculated by dividing shuffled times by the number that was greater or equal to the true prediction correlation. All *P* values estimated by the permutation test were under 0.05.

Because of nonoverlapping participants in the internal and external validation group, we evaluated the *P* value between the observed and predictive *Gf* scores using parametric statistical analysis only^1^.

### Predictive model for external validation

To construct a predictive model of *Gf* to apply to a completely independent group, 326 participants at time 1 data were used to define consensus features^53^. Consensus features were defined as features that were selected in each LOOCV and were used to construct a predictive model of *Gf*. Next, the strength in the feature positions for each participant was computed in the external validation group. After performing LOOCV, we computed the correlations between observed and predicted *Gf* scores using Pearson’s correlation to evaluate the power of the predictive model.

### Validation analysis of confounding factors

Validation analysis was performed to estimate the influence of confounding factors on predictive power. To better understand the variables in internal and external validation groups, the relationships between observed *Gf* scores and head motion, age, and sex were evaluated post-hoc using Pearson’s correlation analyses. To validate the specificity of the *Gf* predictive model, Pearson’s correlation analyses were also performed between age and head motion and predictive scores. In addition, the other influences of confounding factors on functional connectivity analysis was also considered in the *Gf* predictive model, including partial correlation analysis (controlling for age and sex), GM signals effect in data preprocessing, and group-level WM mask.

## Results

### Internal validation I: prediction from WM functional connectivity at time 1

We first verified that the observed *Gf* scores did not correlate with head micromovements (mean FD) during scanning (internal validation group, time 1: *r*_(324)_ = −0.051, *P* = 0.358; time 2: *r*_(103)_ = 0.095, *P* = 0.468; time 3: *r*_(81)_ = 0.027, *P* = 0.812; external validation group: *r*_(51)_ = −0.028, *P* = 0.841) and age (internal validation group, time 1: *r*_(324)_ = −0.026, *P* = 0.635; time 2: *r*_(103)_ = 0.126, *P* = 0.200; time 3: *r*_(81)_ = 0.315, *P* = 0.112; external validation group: *r*_(51)_ = 0.025, *P* = 0.861) (Fig. S3). We also confirmed no differences in the observed *Gf* scores between females and males using two tailed *t*-tests (internal validation group, time 1: *t*_(324)_ = 1.337, *P* = 0.182; time 2: *t*_(103)_ = 1.661, *P* = 0.100; time 3: *t*_(81)_ = 1.004, *P* = 0.318; external validation group: *t*_(51)_ = 1.124, *P* = 0.266) (Fig. S4).

**Fig. 2:**
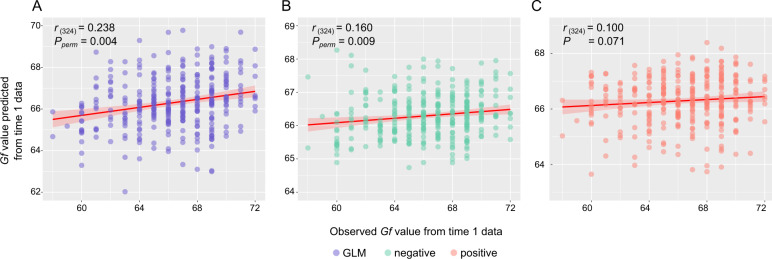
Internal validation I: WM functional connectivity-based predictive models predicted an individual’s *Gf* abilities at time 1. Results from a LOOCV comparing predicted and observed individuals’ *Gf* scores (*n* = 326). Scatter plots showed the predictions of the **a** GLM model (combined the strength of negative and positive features), **b** negative-feature model, and **c** positive-feature model at feature selection of *P* < 0.01 thresholded. Each dot represented each participant. The GLM model and negative-feature model showed significant predictions, and the positive-feature model was in marginal prediction. The negative-feature and positive-feature may provide nonoverlapped information on predicting individuals’ *Gf* abilities. WM white-matter, *Gf* general fluid intelligence, LOOCV leave-one-out cross-validation, GLM general linear model.

After performing the predictive model analyses, we found that WM functional connectivity could be used as a feature to predict individual *Gf* (*r*_(324)_ = 0.238, *P* = 1.44 × 10^−5^) (Fig. 2a). In addition, the *P*_permutation_ was 0.004, reliably suggesting the significant correlation. The power of the predictive model was not affected by head motion, as the features sensitive to head motion were excluded and the predicted *Gf* scores were not correlated with mean FD values (*r*_(324)_ = −0.082, *P* = 0.141) (Fig. S5A). As an observation of the predictive power of negative and positive networks, we also performed the correlation analysis between predicted and observed *Gf* values for the positive-feature and negative-feature models. The negative-feature model also generated significant prediction (*r*_(324)_ = 0.160, *P*_permutation_ = 0.009, Fig. 2b), and the positive-feature model exhibited marginal prediction (*r*_(324)_ = 0.100, *P* = 0.071,, Fig. 2c). The predictions of positive-feature and negative-feature models were not significantly different (Steiger’s *z* value = 0.681, *P* = 0.516; Steiger’s *z* value was obtained from https://www.psychometrica.de/correlation.html), whereas the combined negative and positive features (GLM model) were more accurate than the prediction of the positive-feature model (Steiger’s *z* value = 2.84, *P* = 0.004), and marginally more accurate than the prediction of the negative-feature model (Steiger’s *z* value = 1.38, *P* = 0.084), suggesting, to some extent, that the negative and positive networks provided some complementary information. Thus, the GLM model was applied to further internal validations and external validation.

Furthermore, the correlation coefficient between observed and predicted *Gf* scores was 0.204 ± 0.013 (mean ± SD) in *k*-fold cross-validation. The significant correlation in the 20-fold cross-validation protocol indicated the rigorous presence of WM functional connectivity in an individual’s *Gf* abilities.

### Internal validation II: prediction from WM functional connectivity at time 2

**Fig. 3:**
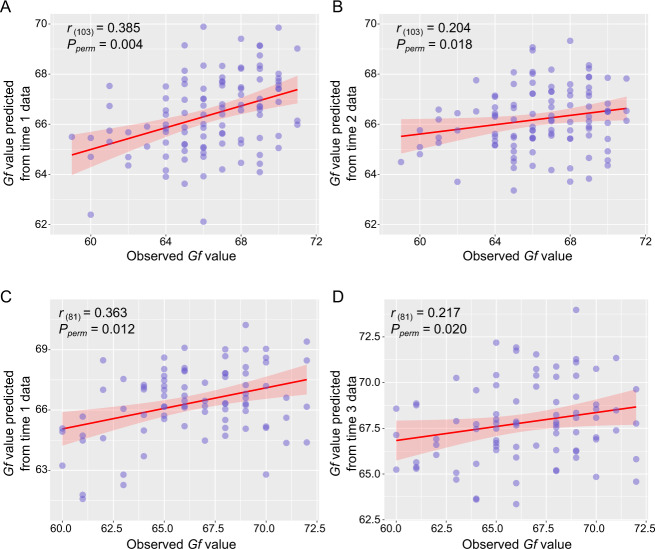
Internal validations II and III: the constructed predictive model of *Gf* at time 1 was generalized to time 2 (time 1 ∩ time 2: *n* = 105) and time 3 (time 1 ∩ time 3: *n* = 83). Scatter plots show correlations between observed and predicted *Gf* abilities. **a**, **b** WM functional network models were iteratively trained on time 1 data from *n* − 1 participants (*n* = 105) and tested on time 1 and time 2 data from the left-out individual. **c**, **d** Similarly, the WM functional network model was also iteratively trained on time 1 data from *n* − 1 participants (*n* = 83) and tested on time 1 and time 3 data from the left-out individual. There was no difference between the two powers of the predictive models from WM connectivity matrixes at time 1 and time 2 (Steiger’s *z* value = 1.773, *P* = 0.076) and time 1 and time 3 (Steiger’s *z* value = 1.163, *P* = 0.244). *Gf* general fluid intelligence, WM white-matter.

We next demonstrated that the constructed predictive model of *Gf* at time 1 data were generalized to time 2. To this end, we selected the participants who participated both in time 1 and time 2 scans (*n* = 105). We reconstructed the predictive model of *Gf* described in the above section “Internal validation I: prediction from WM functional connectivity at time 1” at time 1 (*n* = 105). As expected, models trained on 105 participants’ data also significantly predicted *Gf* values at time 1 (*r*_(103)_ = 0.385, *P*_permutation_ = 0.004) (Fig. 3a). To determine the generalization performance of the predictive model of *Gf* in the same group, we constructed the WM functional connectivity at time 2. We used the same procedure to predict an individual’s *Gf* scores as was done at time 1, except that the test data was from the WM functional connectivity matrix at time 2.

We demonstrated that the training model, based on WM functional connectivity at time 1, predicted an individual’s *Gf* scores from the WM connectivity matrix at time 2. The significant correlation parameters between the observed *Gf* scores and predicted *Gf* scores were *r*_(103)_ = 0.204, *P*_permutation_ = 0.018 (Fig. 3b). Although the correlation coefficient was numerically attenuated at time 2, the powers of the predictive model, from WM connectivity matrices at time 1 and time 2, showed no difference (Steiger’s *z* value = 1.773, *P* = 0.077). These results indicated, within WM functional connectivity, that there was a basis for neural correlates of individual *Gf* abilities and also suggested reliability of the overall model. This effect also could not be explained by head motion, as the predicted *Gf* scores from time 2 were not correlated with mean FD values (*r*_(103)_ = −0.009, *P* = 0.930) (Fig. S5B).

### Internal validation III: prediction from WM functional connectivity at time 3

To further explore whether the predictive model was still stable after a longer fMRI scanning (~29 months interval), we reconstructed the predictive model of *Gf* based on 83 participants’ fMRI data at time 1 (time 1 ∩ time 3: *n* = 83). The strength of WM functional connectivity also significantly predicted *Gf* values (*r*_(81)_ = 0.363, *P*_permutation_ = 0.012, Fig. 3c). This trained model was applied to the WM connectivity matrix at time 3. Significant correlations emerged between predicted and observed *Gf* scores (*r*_(81)_ = 0.217, *P*_permutation_ = 0.020, Fig. 3d). Although the correlation coefficient between predicted and observed *Gf* values at time 3 was numerically smaller than at time 1, the predictive power showed no difference (Steiger’s *z* value = 1.163, *P* = 0.244). The predictions further indicated the reliable neuromarker of *Gf* from WM functional connectivity. The power of this predictive model was not affected by head motion, thus excluding the head motion confounding factor (time 3: *r*_(81)_ = −0.019, *P* = 0.866) (Fig. S5C). In addition, we have also constructed a full correlation matrix across times 1, 2, and 3 for observed and predicted *Gf* scores. We also found that the observed *Gf* score correlated with predicted *Gf* scores in GLM (Table S2). Collectively, these results suggested that although, to a certain degree, brain functional connectivity patterns were metastable or unstable during short or long-time development, the *Gf* predictive model from WM functional connectivity was relatively stable, and did not change across longitudinal scanning times (time 1, time 2, and time 3).

### External validation: prediction at an independent sample from another center

**Fig. 4:**
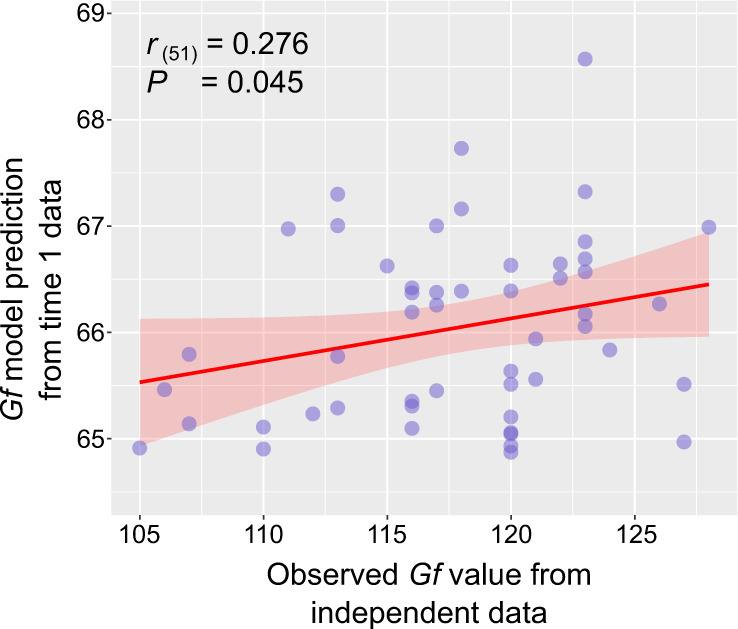
External validation: Gf model, defined with time 1 participants (*n* = 326), significantly predicted an individual’s Gf abilities in a completely independent sample group (*n* = 53). *Gf* general fluid intelligence.

In order to generalize the predictive model of *Gf* to a completely independent data set, we entered positive and negative values into the predictive model to predict individual *Gf* scores. The observed *Gf* scores were marginally correlated with predicted *Gf* scores in the independent sample group (*r*_(51)_ = 0.276, *P* = 0.045, Fig. 4). The correlation coefficient (*r*) was relatively small, but this value was typically between 0.2 and 0.5, according to the previous studies^1,54^. Prediction results will often have lower within-sample effect sized than results generated with simple correlation analysis^1^. Similarly, predicted *Gf* scores were not correlated with mean FD values, ruling out the head motion confounding factor (*r*_(51)_ = −0.169, *P* = 0.228, Fig. S5D).

### Validations of the effects of confounding factors

To validate the specificity of the *Gf* predictive model, we investigated the correlation between predicted *Gf* scores and confounding factors. There were no significant correlations between predicted *Gf* scores and age in internal and external validation groups (time 1: *r*_(324)_ = −0.008, *P* = 0.884; time 2: *r*_(103)_ = 0.086, *P* = 0.384; time 3: *r*_(81)_ = −0.086, *P* = 0.441; independent sample group: *r*_(51)_ = −0.116, *P* = 0.409) (Fig. S5E–H). Otherwise, we performed partial correlation analyses controlling for age and sex in the *Gf* predictive model^1,46,54^, which resulted in significant correlations between observed and predicted *Gf* scores at time 1 (*r*_(324)_ = 0.211, *P* = 1 × 10^−4^, Fig. S6). To further examine the GM signals effect on our results^27^, we regressed out the global brain signals (including GM, CSF, and WM signals) in the neuroimaging data preprocessing and maintained all other processes. We observed that the predicted *Gf* scores were also correlated with the observed *Gf* scores (*r*_(324)_ = 0.217, *P* < 1 × 10^−4^, Fig. S7). Finally, we created a new WM mask across all participants to validate the parcellation effect on our results. We used time 1 data as the mask validation sample, and found that the predictive power still remained at time 1 data (*r*_(324)_ = 0.207, *P* = 2 × 10^−4^, Fig. S8). The two predictive models based on different masks showed no difference on predictive power (Steiger’s *z* value = 0.581, *P* = 0.561). These results suggested that the *Gf* predictive model based on WM functional connectivity could identify relevant *Gf* attributes rather than other confounding factors.

### Functional locations of consensus features on WM connectome

**Fig. 5:**
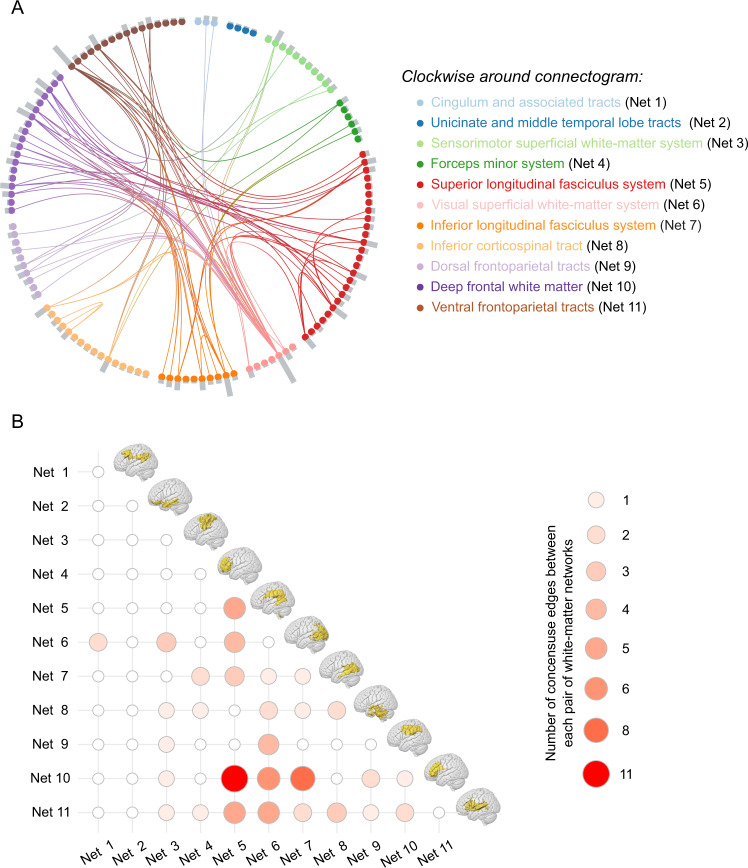
WM functional edges predicts an individual’s *Gf* abilities. **a** The location of consensus features. We divided the 128-ROI in WM into 11 WM functional networks. Macroscale regions were mainly located in the superior longitudinal fasciculus, deep frontal white-matter, and ventral frontoparietal tracts. The gray column in each node represented the degree of this node. **b** The differences in the number of edges in the WM functional networks. *Gf* fluid intelligence, ROI region of interest; WM white-matter.

To illustrate the biological substrates underlying the neural correlates of individual *Gf* and WM functional connectivity, we described the most specific edges (i.e., consensus features) contributing to the predictive model^1,53^. We first divided 128 nodes into 11 specific WM networks (considered as mask) based on a previous study on human WM functional networks (Fig. 5a). We then examined the within- and between-WM functional connectivity consensus features. Although these features were generated from data-driven analyses that were not dependent on prior acknowledge, the features exhibited specific distributions, and were primarily located between the superior longitudinal fasciculus and deep frontal WM and ventral frontoparietal tracts; the number of connections is shown in Fig. 5b. The involvement of the superior longitudinal fasciculus in WM functional networks suggested that the connections did not provide structural information but provided functional information corresponding to *Gf* scores^6,13^.

## Discussion

We showed that the supplementary neuromarker-WM functional connectivity was both reliable and generalizable to establish brain–behavior relationships. We demonstrated that the WM functional profiles could be used as features to predict the fundamental cognitive traits of individuals’ *Gf* abilities. We used a cross-validation, data-driven analysis to identify the connectivity strength of WM functional networks in predicting *Gf*. This predictive model was generalizable to predict individual *Gf* abilities in the same group at the time of scanning (time 1), 11 months later (time 2), and 29 months later (time 3), providing evidence for stable *Gf* features from WM functional connectivity. To demonstrate more generalization and robustness of the predictive model, we used a completely independent sample group as a test cohort. The features that predicted individual *Gf* abilities at time 1 (*n* = 326 participants) also predicted *Gf* scores in an independent sample. These results that the predictive model from WM functional networks predicted an individual’s *Gf* abilities in disparate groups and with difference assessment scales, underscored the potential to investigate the neuromarkers of cognitive trait from whole-brain WM functional connectivity.

Our WM functional connectome-based predictive model, which was generalized to a completely independent cohort, provides a new insight into investigating neuromarkers related to *Gf* abilities. It is noteworthy that this predictive power accounts for ~7.62% of the explained variance. According to previous protocols of connectome-based predictive modeling^1^, this correlation will typically be between 0.2 and 0.5; because, the predictive power will often have lower within-sample effect sizes than results generated with simple correlation analysis. In addition, the cross independent data validation could provide a more conservative estimate of the strength of the brain–behavior relationship and is more likely to generalize independent data^1^. The model showed that human intelligence was unlikely supported by one brain area or one system, but rather was related to several cognitive components (such as the cognitive control network)^55^. This powerful model was successfully applied to illustrate the relationships between functional connectivity in GM and *Gf* scores^11^ and sustained attention^46^. But these predictive models did not focus on WM functional connectivity in part because of the longstanding controversy regarding the functional role of WM BOLD-fMRI signals.

The first debate relates to the way in which WM, relative to GM, contains lower cerebral blood flow and volume. Cerebral blood flow and volumes are the basis of the BOLD signal in neural activity^56–58^. Another debate centers on how BOLD signals recognize local field action potentials in GM, but not in WM^16,59^. However, in comparison with GM, and regardless of large discrepancies in respect to the physiological factors observed between GM and WM, WM maintains a higher ratio of glial cells to neurons^60^, and has an approximately equal oxygen extraction fraction^61^. Many studies identifying WM signals in resting-state fMRI have been reported^16,25,26,29^, suggesting that there are no fundamental barriers or direct sources of evidence against the possibility of detecting WM neural activities using BOLD-fMRI^16^. Furthermore, our previous study has proven that several confounding factors (i.e., CSF signals, global brain signals, and physical distance) did not influence the small-worldness property of WM functional networks^27^.

In addition to showing that the WM connectivity-based predictive model was able to predict *Gf* abilities, the current results also suggested that the distributed networks relevant to *Gf* support partial classical P-FIT characterizing human intelligence. P-FIT describes the interactions of distributed networks in frontal and parietal regions in order to predict the differences in an individual’s *Gf* abilities^13^. The P-FIT model summarizes five steps for information processing of *Gf*^13^. First, salient information is mainly collected by auditory and visual methods, thus, occipital and temporal regions are critical to sensory information processing. Second, parietal regions mainly in the supramarginal, angular, and superior parietal gyrus extract perceptual information. Third, the assumption of interactions between parietal and frontal regions is used to test the hypotheses. Next, the anterior cingulate cortex is used to select and inhibit responses. Finally, the speedy and error-free information processing from the parietal to frontal regions is dependent on WM structures, such as the superior longitudinal fasciculus^6^. Our results have added to the P-FIT model in that functional information in the superior longitudinal fasciculus system is a basis for neural correlates with *Gf*. Other regions related to *Gf* mainly included the visual superficial WM system; deep frontal WM (including the cingulum); the ventral frontoparietal tracts; and the forceps minor system^29^. Several of these WM functional systems are correlated with GM networks, such as the visual superficial WM system, which itself is highly correlated with visual GM networks. Our finding regarding a direct relationship between these WM functional networks and *Gf* is in agreement with steps 1–4 in P-FIT information processing. However, there were other WM functional networks, including the sensorimotor and ventral and dorsal attention GM networks, that showed high correlation with *Gf*, which were not included in the P-FIT model. Thus, our results demonstrated the importance of whole-brain imaging to investigate an individual’s *Gf* abilities.

The specific distributions of relevant edges associated with *Gf* may indicated that BOLD-fMRI signals in WM were nonartifactual and nonrandom noises. Otherwise, the excluded possibility of observed WM functional connectivity from random noise can be validated by the fact that the predictive model is generalized to an independent data set. In this study, we also applied several methods to ensure that WM BOLD-fMRI signals were not affected by GM signals, by strictly controlling the boundary between WM and GM (i.e., we employed a 90% threshold on the WM probability map); by separating WM and GM functional images in preprocessing^26,29^; and by identifying participants’ voxels only in WM to create WM masks. Moreover, from the architecture of the brain venous systems, the possibility that deoxygenated blood was drained from cortical GM to deep WM was rather small. In fact, there are two venous systems in normal neuroanatomy: one is the superficial venous system, which drains deoxygenated blood in GM and superficial WM in the cortex into the pial veins; the other is deep system draining deoxygenated blood in deep WM into the subependymal veins^19,62^. The venous architecture does not overlap in brain regions. Deoxygenated blood drainage from GM cortex to the deep venous system through the WM does exist, but the probability of draining is less than 3%^19,62^. Collectively, these methods, and the brain venous system architecture, ensured that BOLD-fMRI signals analyzed herein were in fact from WM.

Although our findings provided a novel WM connectivity-based predictive model to predict an individual’s *Gf* abilities, several limitations in this study remain. First, the sample in this study was comprised of college students, limiting the application of WM functional connectome-based predictive model for other populations. Future studies should employ a wider population base to investigate the relationship between *Gf* and WM functional connectomes. Second, individuals’ *Gf* abilities were assessed by the CRT in this study. Although the completely independent sample from another center was assessed by WAIS-RC, given the various types of psychometric tests for human intelligence, future studies should further test the specificity and generalization of the current predictive model using a wide variety of intelligence measures to detect the consensus neuromarker of intelligence in WM. Third, the current work used WM functional connectivity as a neuromarker, ignoring the causality connectivity (effective connectivity) among the brain regions. Future effective connectivity studies on WM functions are therefore needed. Finally, the physiological basis of BOLD-fMRI signals in WM remains unknown. However, a previous study suggested that the impact of artifacts (such as physiological noise and head motion) in WM activity is relatively small^16,26^. In this study, the possible effects of artifacts were strictly controlled by exclusion of excessive head motion, regression of CBF signals, regression of global brain signals, and exclusive preprocessing of WM. Future neurophysiological studies on BOLD-fMRI signals in WM are needed to more accurately interpret the current predictive model.

In conclusion, we demonstrated that functional connectivity in WM was a neuromarker to predict an individual’s *Gf* abilities and that the distributed networks supported the P-FIT model. Beyond the current findings, the predictive model from whole-brain functional connectivity in WM can be used to investigate other cognitive abilities and clinical symptoms in psychiatric diseases.

## Supplementary information

SUPPLEMENTAL MATERIAL

## Data Availability

The code of this predictive model was provided by Shen et al. at https://www.nitrc.org/projects/bioimagesuite/.
