# Supplementary material for: A neuromarker of individual general fluid intelligence from the white-matter functional connectome
Source: Transl Psychiatry. 2020 May 13;10:147. doi: 10.1038/s41398-020-0829-3 (PMC7220913; doi:10.1038/s41398-020-0829-3)
Supplement: Supplementary file 1 — SUPPLEMENTAL MATERIAL [file 41398_2020_829_MOESM1_ESM.pdf]

Supporting Information for

A neuromarker of individual general fluid intelligence from the white-matter functional connectome

Running title: Intelligence and WM functional connectome

Jiao Li[a,b], Bharat B. Biswal[a,b,c], Yao Meng[a,b], Siqi Yang[a,b], Xujun Duan[a,b], Qian Cui[a,b,d], Huafu Chen[a,b]*, Wei Liao [a,b]*

[a] The Clinical Hospital of Chengdu Brain Science Institute, School of Life Science and Technology, University of Electronic Science and Technology of China, Chengdu 610054, P.R. China.

[b] MOE Key Lab for Neuroinformation, High-Field Magnetic Resonance Brain Imaging Key Laboratory of Sichuan Province, University of Electronic Science and Technology of China, Chengdu 610054, P.R. China.

[c] Department of Biomedical Engineering, New Jersey Institute of Technology, Newark, NJ 07102, USA.

[d] School of Public Administration, University of Electronic Science and Technology of China, Chengdu 610054, P.R. China.

* Corresponding authors:

Huafu Chen (chenhf@uestc.edu.cn), and Wei Liao (weiliao.wl@gmail.com),

The Clinical Hospital of Chengdu Brain Science Institute, MOE Key Laboratory for Neuroinformation,

School of Life Science and Technology,

University of Electronic Science and Technology of China,

Chengdu 610054, P.R. China.

CONTENTS

Supplementary Materials and Methods 3

External validation 3

Participants 3

Behavioral assessment 3

Neuroimaging data acquisition and preprocessing 3

Construction of WM functional connectome 4

Supplementary Figures 5

Figure S1 5

Figure S2 6

Figure S3 7

Figure S4 8

Figure S5 9

Figure S6 10

Figure S7 11

Figure S8 12

Supplementary Tables 13

Table S1. Demographics for time 1, time 2 and time 3 samples. 13

Table S2. Full correlation matrix across times 1, 2 and 3 for observed and predicted *Gf* scores. 14

References 15

Supplementary Materials and Methods

External validation

Participants

This study was approved by the Ethics Committee of the Shanxi Medical University, Shanxi, China. Written informed consent was obtained from all participants. A total of 78 normal controls (55 females, mean age = 26.82 years, SD = 6.88 years) were recruited. All participants were no psychiatric and neurological disorders, and have no history of substance, drug, or alcohol dependence.

Behavioral assessment

Participants' intellectual ability was assessed by the Chinese version of the Wechsler Adult Intelligence Scale (WAIS-RC) [1]. WAIS-RC is composed by two sets, including verbal intelligence quotient (VIQ) test and performance intelligence quotient (PIQ) test. VIQ test includes six subtests: Information, digit Span, Vocabulary, Arithmetic, Comprehension and Similarities. PIQ test includes five subtests: Picture Completion, Picture Arrangement, Block Design, Object Assembly and Digit Symbol. WAIS-RC measures fluid intelligence on the performance scale, while crystallized intelligence on the verbal scale [2]. To keep the IQ dimension with the *Gf*, we chose PIQ values to measure the independent samples' intelligence abilities.

Neuroimaging data acquisition and preprocessing

Imaging data were acquired using a 3-T MRI scanner (Magnetom Trio, Siemens Healthcare, Germany) located at Shanxi Provincial People's Hospital, Shanxi, China. Both 3D T1-weighted structural images and resting-state fMRI were acquired. During resting-state fMRI scanning, all participants were instructed simply to rest with their eyes closed, and not to think of anything in particular. Structural images were obtained using the following parameters: repetition time = 2300ms, echo time = 2.95ms, inversion time = 900ms, flip angle = 9 degrees, matrix = 240 × 240, slices = 160, thickness = 1.2mm, voxel size = $1.2 \times 0.94 \times 0.94 mm^3$. Resting-state fMRI was obtained

by: slice = 32, repetition time/echo time = 2500/30ms, flip angle = 90 degrees, thickness = 3mm, gap = 1mm, matrix size = 64 × 64, voxel size = 3.75 × 3.75 × $3mm^3$, and a total of 212 volumes for each participant.

Data were analyzed using the DPARSF (v4.3, www.restfmri.net) and SPM12 toolkits (www.fil.ion.ucl.ac.uk/spm/software/spm12). Slice-timing correction and realignment were applied to the remaining 205 functional images after excluding the first seven images. All next steps were the same as internal validation group preprocessing.

According to the exclusion criteria in internal validation, 25 participants were excluded from the sequent analysis, including 13 participants with excessive head motion (mean FD > 0.15, rotation > 2°, translation > 2mm, or participant's points on an image were < 80% after performing scrubbing analysis), seven subjects with low quality of functional images, and five subjects defined as outliers. Finally, 53 subjects (41 females, mean age = 24.98 years, SD = 2.54 years) were left as external validation group.

Construction of WM functional connectome

Network regions were defined using a subset of nodes of the 128-random parcellation [3] or the network analysis in internal validation samples [4]. As some scans did not include cerebellum, brainstem and temporal poles coverage, five participants missing at least five nodes were removed. The five participants were included in above-mentioned low quality of functional images. All constructing WM functional connectivity were identical to those described in the internal validation analysis.

Supplementary Figures

Figure S1

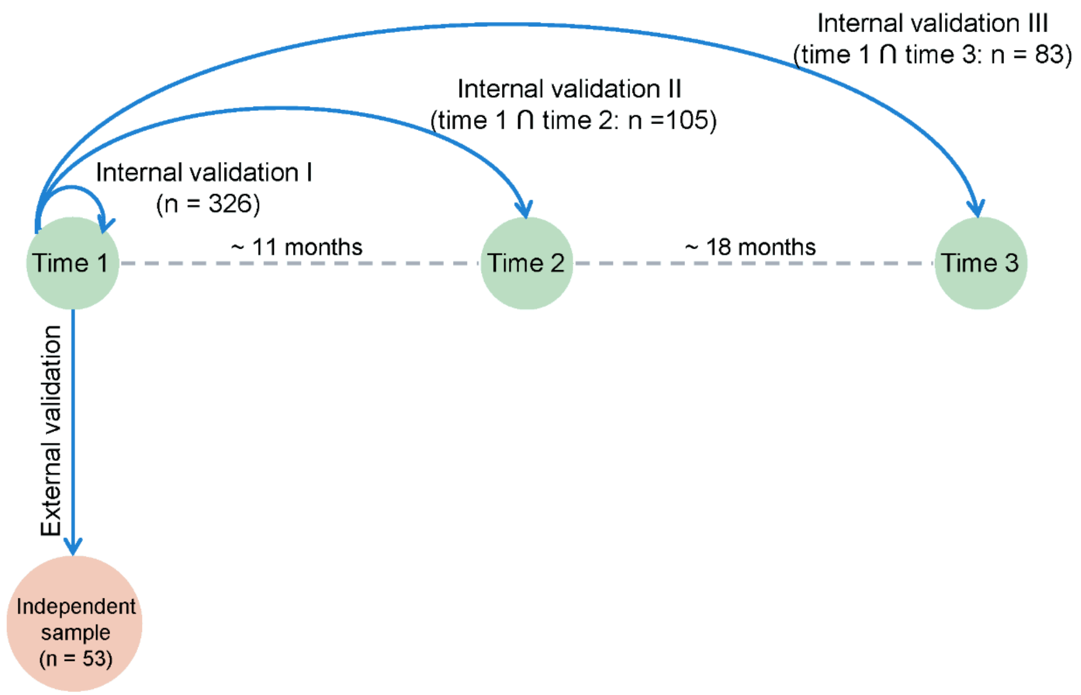

Figure S1. Overview of the current study. The number of participants in this figure was obtained after quality control. The current study included internal and external validation groups. Three interval validation groups are: Internal validation I (time 1: n = 326) was trained on time 1 data and also tested on time 1 data using Leave-one-out cross-validation (LOOCV). Internal validation II (time 1 ∩ time 2: n = 105) included trained on time 1 data (n–1 participants, n = 105) and tested on time 1 and time 2 data (appropriately 11 months after time 1 scanning). Internal validation III (time 1 ∩ time 3: n = 83) included trained on time 1 data (n–1 participants, n = 83) and tested on time 1 and time 3 data (appropriately 29 months after time 1 scanning). For external validation, the predictive model constructed from time 1 data (n = 326) was used to predict a completely independent group (n = 53).

Figure S2

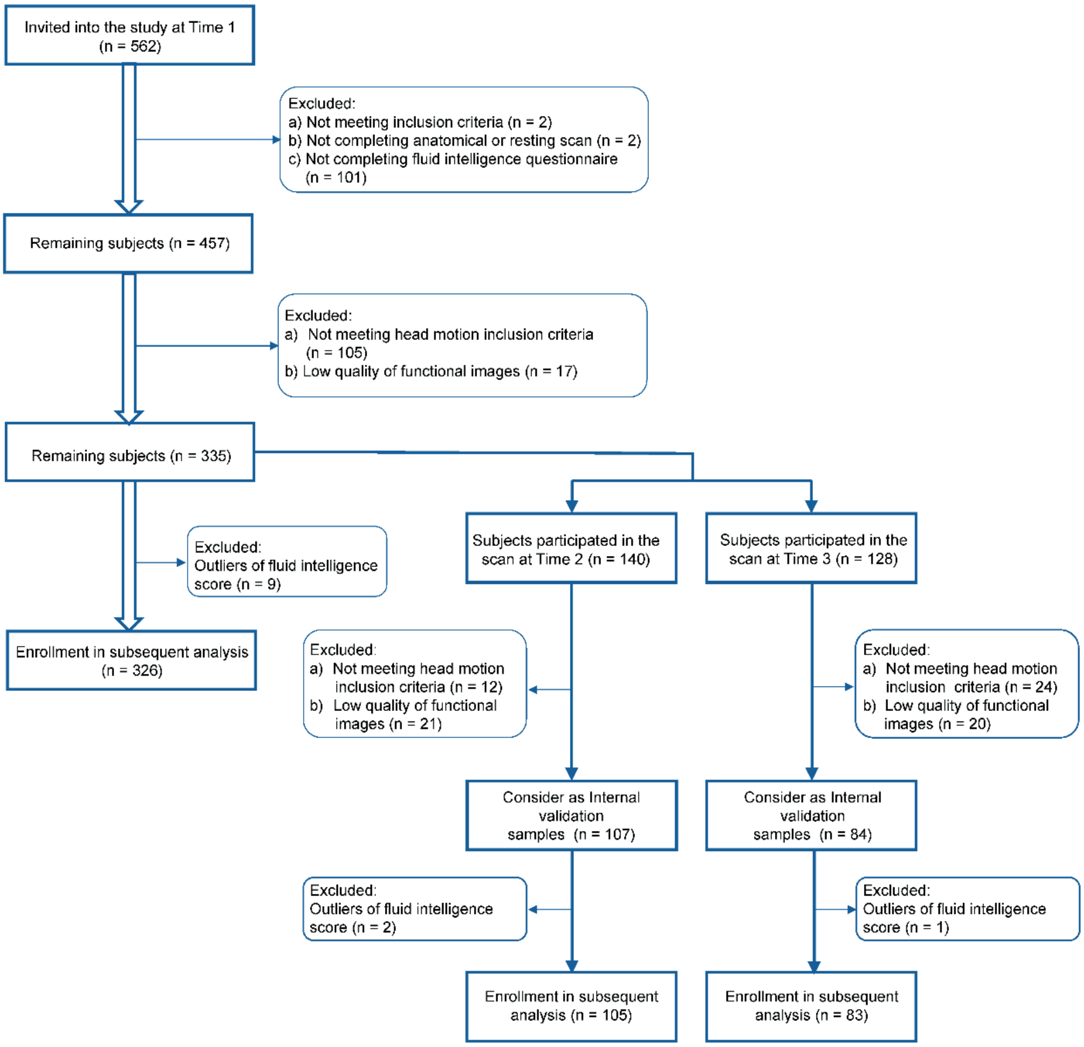

Figure S2. Flow diagram for participant inclusion process. The current study included 326 subjects for internal validation I section, 105 subjects for internal validation II section and 83 subjects for internal validation III section.

Figure S3

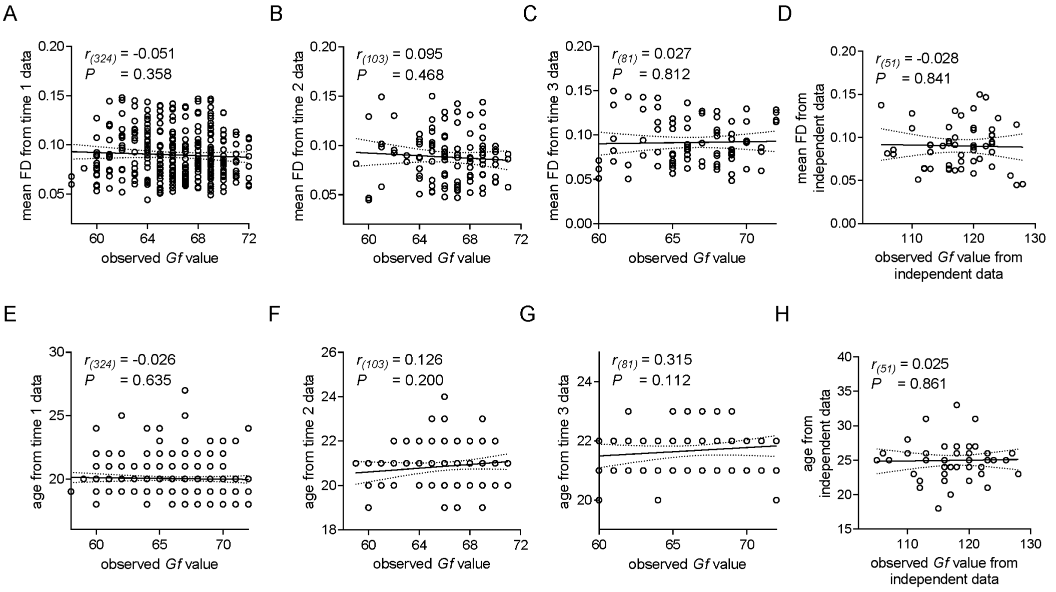

Figure S3. Correlations between observed *Gf* value and mean FD and age from internal and external validation groups. Given that the confound factors (age and mean FD) analyses of WM functional connectivity, we first confirmed that observed *Gf* scores were not correlated with mean FD and age at time 1 data (A & E), time 2 data (B & F), time 3 data (C & G) and independent data (D & H). The solid line and dashed lines represent the best-fit line and 95% confidence interval of Pearson correlation, respectively. Abbreviations: *Gf*, general fluid intelligence; FD, framewise-displacement; WM, white-matter.

Figure S4

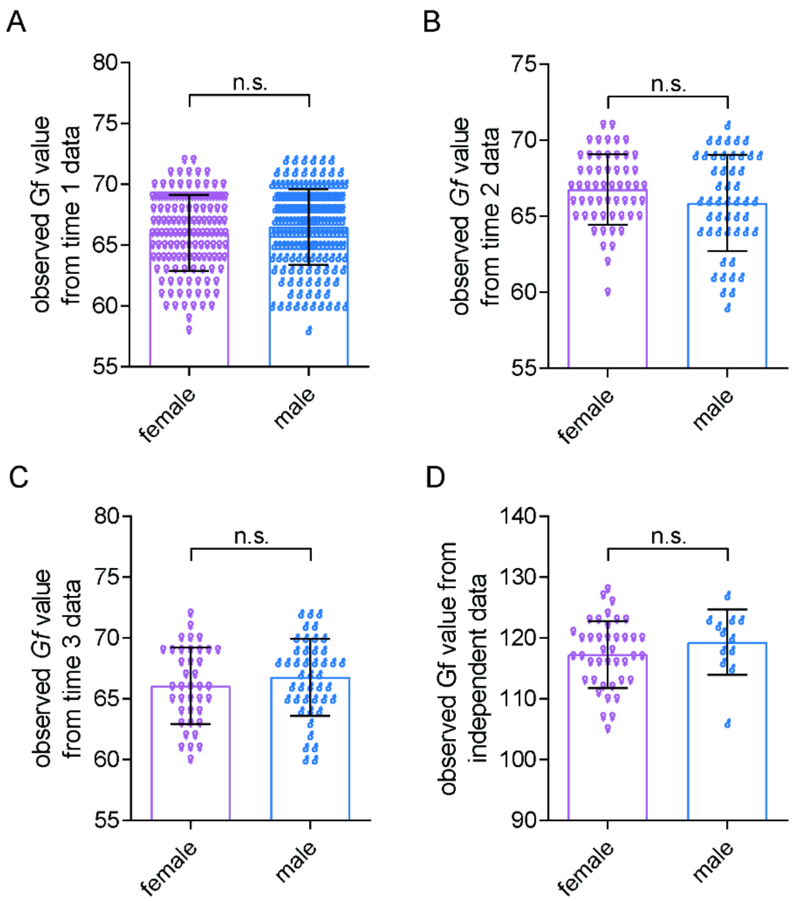

Figure S4. The observed *Gf* value differences between female and male from time 1, time 2, time 3 and independent data. To examine whether the *Gf* values were different in female and male participants, we performed two-sample t-test analysis on time 1 data, time 2 data, time 3 data and independent data independently. No differences of *Gf* values were found between females and males. Abbreviations: *Gf*, general fluid intelligence.

Figure S5

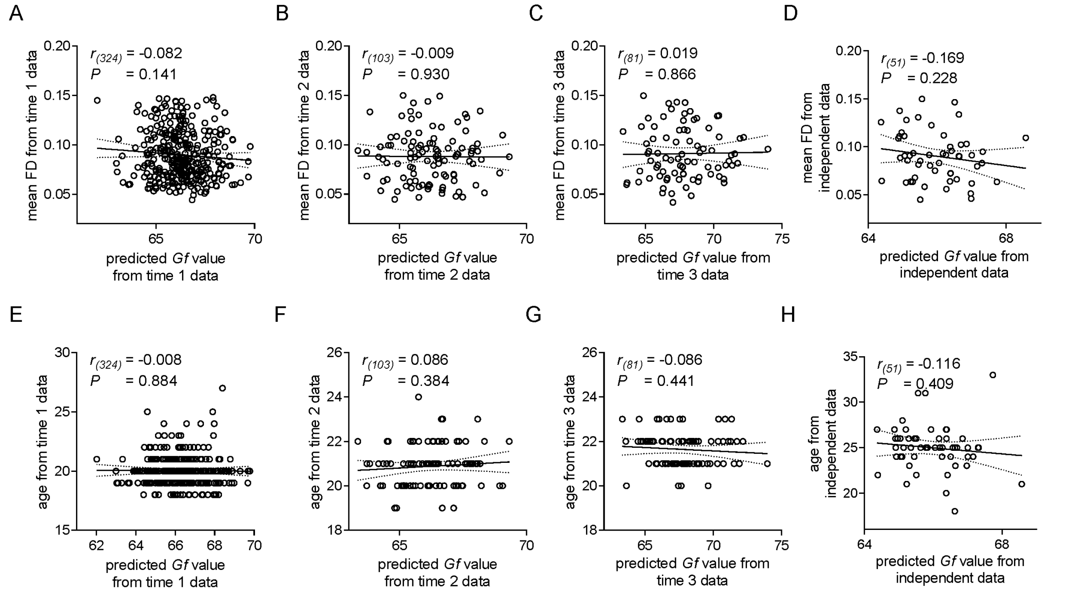

Figure S5. Correlations between predicted *Gf* value and mean FD and age from time1, time 2, time 3 and independent data. To confirm that the predictive model of *Gf* was specific to intelligence abilities, we examined the relationship between predicted *Gf* values and mean FD and age. The results revealed that the predicted *Gf* scores were not correlated with mean FD and age on all groups (time 1: A & E; time 2: B & F; time 3: C & G; independent data: D & H). The solid line and dashed lines represent the best-fit line and 95% confidence interval of Pearson correlation, respectively. Abbreviations: *Gf*, general fluid intelligence; FD, framewise-displacement.

Figure S6

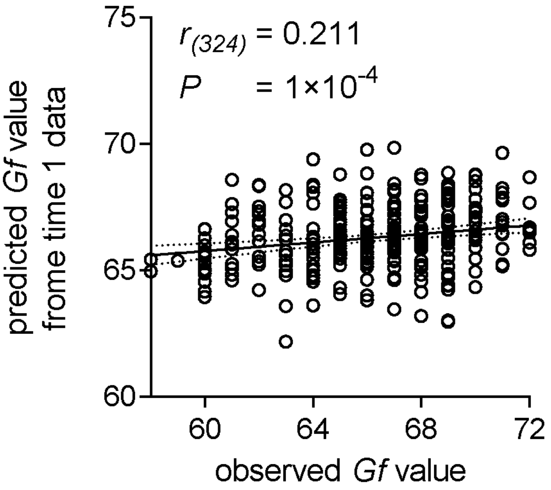

Figure S6. Partial correlations between observed and predicted *Gf* value on time 1 data. To minimize possible confounding effects, we performed partial correlation in feature selection to examine whether brain-behavior relationship was robust to age, sex and FD. The result revealed that the predicted model of *Gf* still has prediction power. The solid line and dashed lines represent the best-fit line and 95% confidence interval of Pearson correlation, respectively. Abbreviations: *Gf*, general fluid intelligence; FD, framewise-displacement.

Figure S7

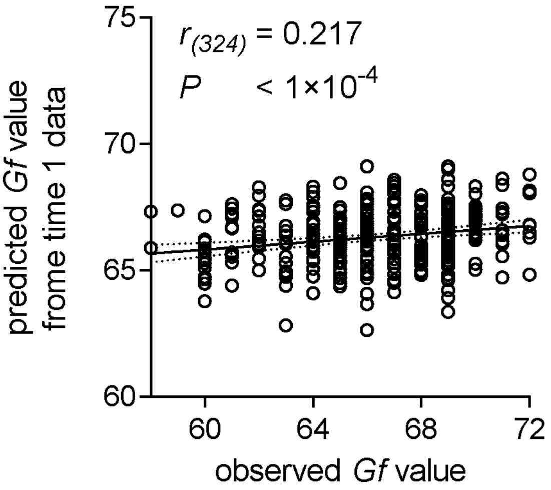

Figure S7. Correlations between observed and predicted *Gf* values on time 1 data after regressing out global brain signals. Although we strictly defined WM mask in our study, we still regressed out global brain signals (including GM, WM and CSF) and maintained all other processes to validate the global signals effect on the prediction model. The results revealed that the power of predictive model remained unchanged. The solid line and dashed lines represent the best-fit line and 95% confidence interval of Pearson correlation, respectively. Abbreviations: *Gf*, general fluid intelligence; GM, gray-matter; WM, white-matter; CSF, cerebrospinal fluid.

Figure S8

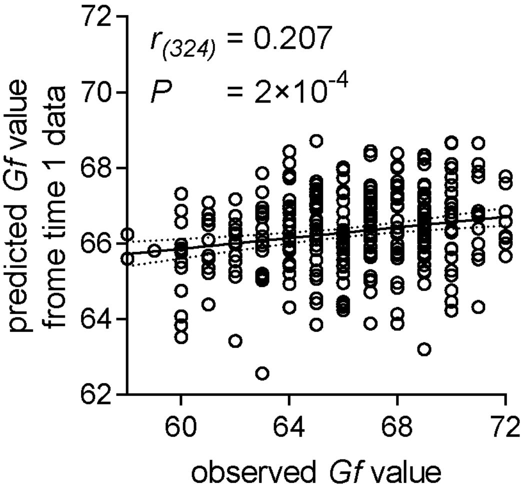

Figure S8. Predictive power of time 1 data after reconstructing WM functional correlation matrix. To examine the effect of WM mask constructing on our predictive model, we reconstructed WM mask across all participants. After reconstructing WM functional connectivity, we found that the predictive *Gf* values also showed significant correlation with observed *Gf* values at time 1 data. And the two predictive models based on different masks showed no difference on predictive power (Steiger's *z* value = 0.581, P = 0.561). The solid line and dashed lines represent the best-fit line and 95% confidence interval of Pearson correlation, respectively. Abbreviations: *Gf*, general fluid intelligence; FD, framewise-displacement.

Supplementary Tables

Table S1. Demographics for time 1, time 2 and time 3 samples.

	Time 1 (n = 326)	Time 2 (n = 105)	Time 3 (n =83)
Age at scan	20.02 (1.29)	20.88 (0.93)	21.67 (0.80)
Sex (female/male)	184/142	49/56	44/39
Gf score	66.29 (3.13)	66.35 (2.76)	66.45 (3.15)

Note: *Gf* scores were obtained at time 1. Data are presented as means (standard deviations).

Table S2. Full correlation matrix across times 1, 2 and 3 for observed and predicted *Gf* scores.

	GLM model	Negative feature	Positive feature
r value	0.374	0.290	0.038
p_{perm} value	0.019	0.073	0.819

Note: GLM, general linear model. GLM combined negative and positive features for predicting *Gf* scores.

References

1 Dai, X. Y., Gong, Y. X. & Zhong, L. Factor analysis of the mainland Chinese version of the Wechsler Adult Intelligence Scale. *A Journal of Consulting and Clinical Psychology* **2**, 31-34 (1990).

2 Wechsler, D. Wechsler Adult Intelligence Test-revised manual. San Antonio, TX: The Psychological Corporation, 1981

3 Li, J. et al. Exploring the functional connectome in white matter. *Hum Brain Mapp* **40**, 4331-4344 (2019).

4 Zalesky, A. et al. Whole-brain anatomical networks: does the choice of nodes matter? *Neuroimage* **50**, 970-983 (2010).
